# Complications of CT-guided lung biopsy with a non-coaxial semi-automated 18 gauge biopsy system: Frequency, severity and risk factors

**DOI:** 10.1371/journal.pone.0213990

**Published:** 2019-03-18

**Authors:** Amany Saad Elshafee, Annika Karch, Kristina I. Ringe, Hoen-oh Shin, Hans-Jürgen Raatschen, Nermin Yehia Soliman, Frank Wacker, Jens Vogel-Claussen

**Affiliations:** 1 Institute of Diagnostic and Interventional Radiology, Hannover Medical School, Hannover, Germany; 2 German Centre for Lung Research, Hannover, Germany; 3 Institute of Diagnostic and Interventional Radiology, Faculty of Medicine Mansoura University, Mansoura, Egypt; 4 Institute for Biostatistics, Hannover Medical School, Hannover, Germany; Michigan Medicine, University of Michigan, UNITED STATES

## Abstract

**Objectives:**

To evaluate frequency and severity of complications after CT-guided lung biopsy using the Society of Interventional Radiology (SIR) classification, and to assess risk factors for overall and major complications.

**Materials and methods:**

311 consecutive biopsies with a non-coaxial semi-automated 18 gauge biopsy system were retrospectively evaluated. Complications after biopsy were classified into minor SIR1-2 and major SIR3-6. Studied risk factors for complications were patient-related (age, sex and underlying emphysema), lesion-related (size, location, morphologic characteristic, depth from the pleura and histopathology), and technique-related (patient position during procedure, thoracic wall thickness at needle path, procedure time length and number of procedural CT images, number of pleural passes, fissure penetration and needle-to-blood vessel angle). Data were analyzed using logistic and ordinal regression.

**Results:**

Complications were pneumothorax and pulmonary hemorrhage. The complications were minor SIR1-2 in 142 patients (45.6%), and major SIR3-4 in 25 patients (8%). SIR5-6 complications were not present. Emphysema, smaller deeply located lesion, increased puncture time length and number of procedural CT images, multiple pleural passes and fissure puncture were significant risk factors for complication severity in univariate analysis. Emphysema (OR = 8.8, p<0.001), lesion depth from the pleura (OR = 1.9 per cm, p<0.001), and fissure puncture (OR = 9.4, p = 0.01) were the independent factors for major complications in a multiple logistic regression model. No statistical difference of complication rates between the radiologists performing biopsies was observed.

**Conclusions:**

Knowledge about risk factors influencing complication severity is important for planning and performing CT-guided lung biopsies.

## Introduction

Transthoracic CT-guided lung biopsy is a minimally invasive procedure for the characterization of pulmonary lesions. It is employed if other techniques, like transbronchial biopsies, are not feasible or have failed [1]. However, some complications such as pneumothorax, hemoptysis, and air embolism can arise after biopsies which may need hospitalization [2]. Thus, it is of great clinical importance for radiologists and clinicians to be aware of frequency and severity of post-biopsy complications, and what the possible risk factors for complications are.

The Society of Interventional Radiology (SIR) had developed a standardized terminology for reporting complications after interventional radiology procedures. These complications were classified by outcome into; minor complications SIR1-2, which require no therapy or require overnight admission for observation only, and major complications SIR3-6, which require therapy and variable periods of hospital stay, or permanent adverse sequelae, or even death [3].

Older age, presence of emphysema, smaller deeply located lesion, longer puncture time, multiple pleural passes and fissure puncture were the most commonly risk factors for complications of CT-guided lung biopsy reported by previous studies [4–8], but none of them, to our knowledge, used the SIR classification for complication severity quantification. Furthermore, identifying the risk factors for overall and major complications will help physicians better assess the risks and manage patients undergoing CT-guided lung biopsies.

Thus, the purpose of this study was to evaluate the frequency and severity of complications after CT-guided lung biopsy using the SIR classification and to assess possible risk factors for overall and major complications.

## Materials and methods

This retrospective study was a single Tertiary Centre cohort study. It was approved by the institutional review board at Hannover Medical School.

### Study population

All consecutive CT-guided core lung biopsies recorded in our database from May 2011 to July 2014 were retrospectively analyzed. A total of 335 biopsies were assessed. Data collection was planned a prior. Data were extracted by a subspecialty-trained thoracic radiology attending physician with 15 years of experience and a radiology medical researcher with 5 years of experience. Data extraction form with questions decided a prior was used. Inclusion criteria included patients who underwent CT-guided core lung biopsy with available images and reports. On the basis of these criteria, 5 canceled biopsies either due to non-cooperative patients (n = 3), highly vascular lesion (n = 1), or lesion too near to a large blood vessel (n = 1), were excluded from the analysis due to lack of immediate post-procedural CT scans and follow-up chest radiographs. Fifteen patients underwent two repeated biopsies and two patients underwent three biopsies, for the same lesion in a different session within one month from the initial biopsy, either due to technically failed first biopsy (n = 9) (failure of placement of the biopsy needle tip within the target lesion or inadequate biopsy specimen from the lesion at visual inspection [9]), or the pathological results of the previous biopsy on the same lesion were non-diagnostic (n = 6), or were benign while the possibility of malignancy still existed (n = 4). For each patient with repeated biopsy, only one biopsy was included to the dataset in order to avoid dependent data in the statistical analysis, thus only the last procedure was used. The final study group consisted of 311 CT-guided core lung biopsies (127 women and 184 men; mean age, 62.2 years). 85 biopsies (27.3%) were performed in hospitalized patients.

### Interventional procedure

Written informed consent of CT-guided biopsy was obtained from each patient before the intervention. The biopsies were performed by five interventional radiologists having more than five years of experience in interventional radiology. Pre-procedure complete blood count and coagulation profile were obtained. The biopsy was performed with platelet count at least 50.000/mm^3^, prothrombin time >60%, and partial thromboplastin time ≤1.5 times. Before beginning the procedure, the interventional strategy, especially patient’s position and biopsy pathway were planned using pre-biopsy chest CT images. The intervention was started with skin disinfection and subcutaneous local anesthesia advanced toward the planned pathway using 20ml lidocaine 1% (Xylocitin, Jenapharm, Germany). The biopsy was performed using a non-coaxial semi-automated 18 gauge biopsy system of a 2cm throw length needle (Somatex; Medical Technologies, GmbH, Teltow, Germany). Procedural CT acquisitions were obtained to check that the needle tip reached the target lesion properly. After needle removal, post-biopsy CT images were acquired to detect complications. The patients were instructed to rest without eating for 2 hours after biopsy to reduce the incidence of post-biopsy pneumothorax. In our institution, a follow-up chest PA radiograph was routinely obtained 2 hours after biopsy to assess post-biopsy complications in particular pneumothorax, and if needed, further follow-up radiographs were performed.

### Data analysis

Procedural and post-procedural CT images, as well as patient- and procedure-related information recorded in both our electronic medical record system and radiological reports of biopsy procedures, were retrospectively reviewed for all biopsies.

#### Complications of the procedure

Complications of the procedure, commonly pneumothorax and pulmonary hemorrhage, were assessed using CT scan obtained directly after the intervention as well as follow-up chest PA radiographs after the intervention. The size and severity of pneumothorax was measured on axial post-biopsy CT images as the largest distance of retraction of pulmonary surface, which was classified into, small pneumothorax <2cm, moderate 2-4cm, and large >4cm [10, 11]. Pulmonary hemorrhage was identified as new ground-glass opacity on post-biopsy CT, documented hemoptysis, or bleeding complications necessitating intervention. The severity of pulmonary hemorrhage on post-biopsy CT was graded into; grade 0 as no hemorrhage or needle tract hemorrhage <1cm (as the needle tract hemorrhage <1cm is often expected from our prior experience after CT-guided lung biopsies, was not considered as a complication), grade 1 as needle tract hemorrhage 1-2cm in width, grade 2 as hemorrhage >2cm in width but sublobar, grade 3 as lobar hemorrhage or greater [12, 13]. Other complications were also assessed. Management of post-biopsy complications for each patient was reviewed and period of hospital stay, if the patient required, was calculated. If the patient, who had the biopsy, was already hospitalized because of other causes rather than post-biopsy complications, only management of post-biopsy complications was being taken in consideration on the basis of the retrospective review. Complications were classified according to the Society of Interventional Radiology (SIR) Guidelines into [3, 14]:

No complications SIR0.

Minor complications SIR1-2: Intervention-related complications not requiring treatment SIR1, or requiring overnight admission for observation only SIR2.

Major complications SIR3-6: Intervention-related complications requiring treatment or hospital admission (minor hospitalization<48h SIR3 and prolonged hospitalization>48h SIR4), or permanent adverse sequelae SIR5, or death SIR6.

#### Risk factors for complications

Multiple variables were evaluated as potential risk factors for complications. Patient-related variables included age, sex, and evidence of underlying emphysema on procedural CT images. Lesion-related variables included mean lesion diameter as lesion size, lesion location, lesion morphologic characteristic, lesion depth from the pleura along the needle path, and lesion histopathology. Technique-related variables included patient position during procedure, thoracic wall thickness at needle path, procedure time and images measured as time length and number of procedural CT images (each of 5mm slice thickness) taken for biopsy needle guidance to the lesion until procedure completion and biopsy needle removal, number of pleural passes, fissure puncture (when a fissure was crossed, two additional pleural passes were calculated [15]), and needle-to-blood vessel angle measured as the angle between a line drawn along trajectory of biopsy needle and a large blood vessel just distal to the lesion (if present), either no angle (or no large blood vessel at the needle trajectory) or a relevant angle (angle ≤90°).

### Statistical analysis

All data were fully anonymized before we accessed. Statistical analysis was performed using JMP (SAS statistical software, version 14.0, SAS Institute, Inc; Cary; NC, USA) and SPSS (IBM SPSS version 24.0, SPSS Inc. Chicago, USA). If not stated otherwise, descriptive results of continuous parameters were given as mean (95% confidence interval CI); ordinal and nominal parameters were presented as absolute frequency (%). As a first descriptive analysis, the association between complications measured by SIR classification and continuous parameters was analyzed using analysis of variance (ANOVA), whereas association of SIR complications with ordinal and nominal parameters was assessed using chi-square tests.

A univariate logistic regression analysis was performed in relation to SIR score (no and minor complications SIR0-2 vs. major complications SIR3-6). In case of complete separation of the outcome in any category of a predictor, logistic regression with firth correction was done. A multiple logistic regression model was built from a medical point of view taking into account the significant predictors from univariate analysis causing no statistical difficulties due to multicollinearity. Furthermore, a univariate ordinal regression was performed for SIR0-6 as ordinal response variable to support the results from univariate logistic regression. Results of ordinal regression were presented as a sensitivity analysis in S1 Table since necessary assumptions (of proportional odds) were not fulfilled for some predictors. Odds ratios (OR) with 95% confidence intervals (CI) were presented for logistic (and ordinal) regressions. In the statistical tests, a p value <0.05 was considered significant.

dx.doi.org/10.17504/protocols.io.wvrfe56

## Results

A total of 311 CT-guided core lung biopsies were evaluated. 184 biopsies (59%) were performed in men and 127 (41%) in women. The mean patient age was 62.2 years (95%CI: 60.5–64.0 years). Underlying emphysema was identified in 65 patients (21%).

The mean lesion size was 3.1cm (95%CI: 2.8–3.3 cm). 116 lesions (37%) were subpleural (abutting the pleural surface). The number of pleural passes per biopsy was one in 103 biopsies, two in 126 biopsies, three in 60 biopsies and four or more in 22 biopsies. Table 1 summarizes lesion- and procedure- variables as risk factors for complications of CT-guided lung biopsy.

**Table 1:** Lesion- and procedure- variables as risk factors for complications of CT-guided lung biopsy (n = 311).

Risk factor	Descriptive
**Lesion size** (cm)	3.1(2.8–3.3)
**Lesion lobar location**	
Right upper	59(19%)
Right lower	88(28%)
Right middle	24(8%)
Left upper	79(25%)
Left lower	61(20%)
**Lesion morphologic characteristic**	
Solid	244(79%)
Subsolid	48(15%)
Consolidative	10(3.2%)
Cavitary	9(2.8%)
**Lesion-to-pleura distance** (cm)	1.7(1.5–1.9)
Lesion histopathology*	
Malignant	207(67%)
Benign	65(21%)
Infection	20(6%)
Non-diagnostic	15(5%)
**Patient position**	
Supine	129(42%)
Prone	156(50%)
Lateral	26(8%)
**Thoracic wall thickness** (cm)	4.0(3.8–4.1)
**Procedure time length** (min)	16.0(15.3–16.8)
**Number of procedural CT images**	32.3(30.3–34.4)
**Fissure puncture**	14(5%)
**Needle-to-blood vessel angle**	
No angle	299(96%)
Angle ≤90°	12(4%)

Continuous parameters are displayed as mean (95% confidence interval CI). Ordinal and nominal parameters are displayed in absolute frequency (%).

*Missing histopathology data in 4 cases (1%).

**Fig 1:**
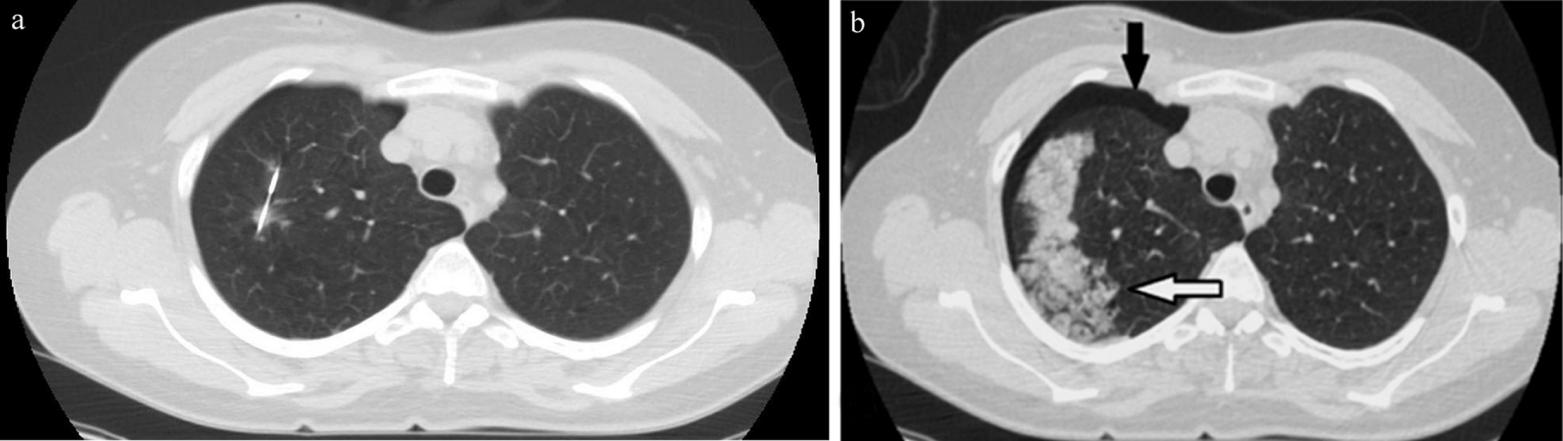
Complications of CT-guided lung biopsy. (a) CT-guided lung biopsy with a non-coaxial semi-automated 18 gauge biopsy system of a 9mm right upper lobe nodule which was proved to be bronchogenic carcinoma at pathologic examination. (b) Post-biopsy CT image showed complications in the form of a small pneumothorax 1cm (black arrow), and a new ground-glass opacity along needle tract measuring >2cm in width but sublobar corresponding to grade 2 pulmonary hemorrhage (white arrow).

**Fig 2:**
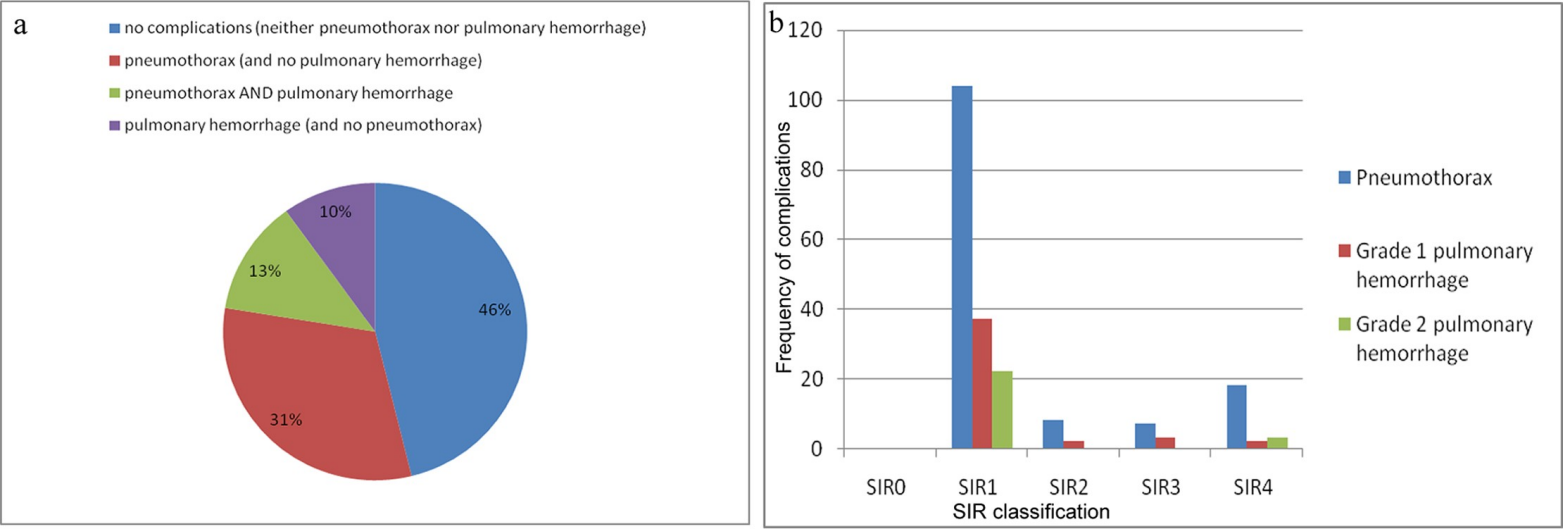
Complications of CT-guided lung biopsy in the study group (n = 311). **(**a) Diagram of percentages of complications. (b) Diagram of frequency of complications in relation to SIR classification.

**Fig 3:**
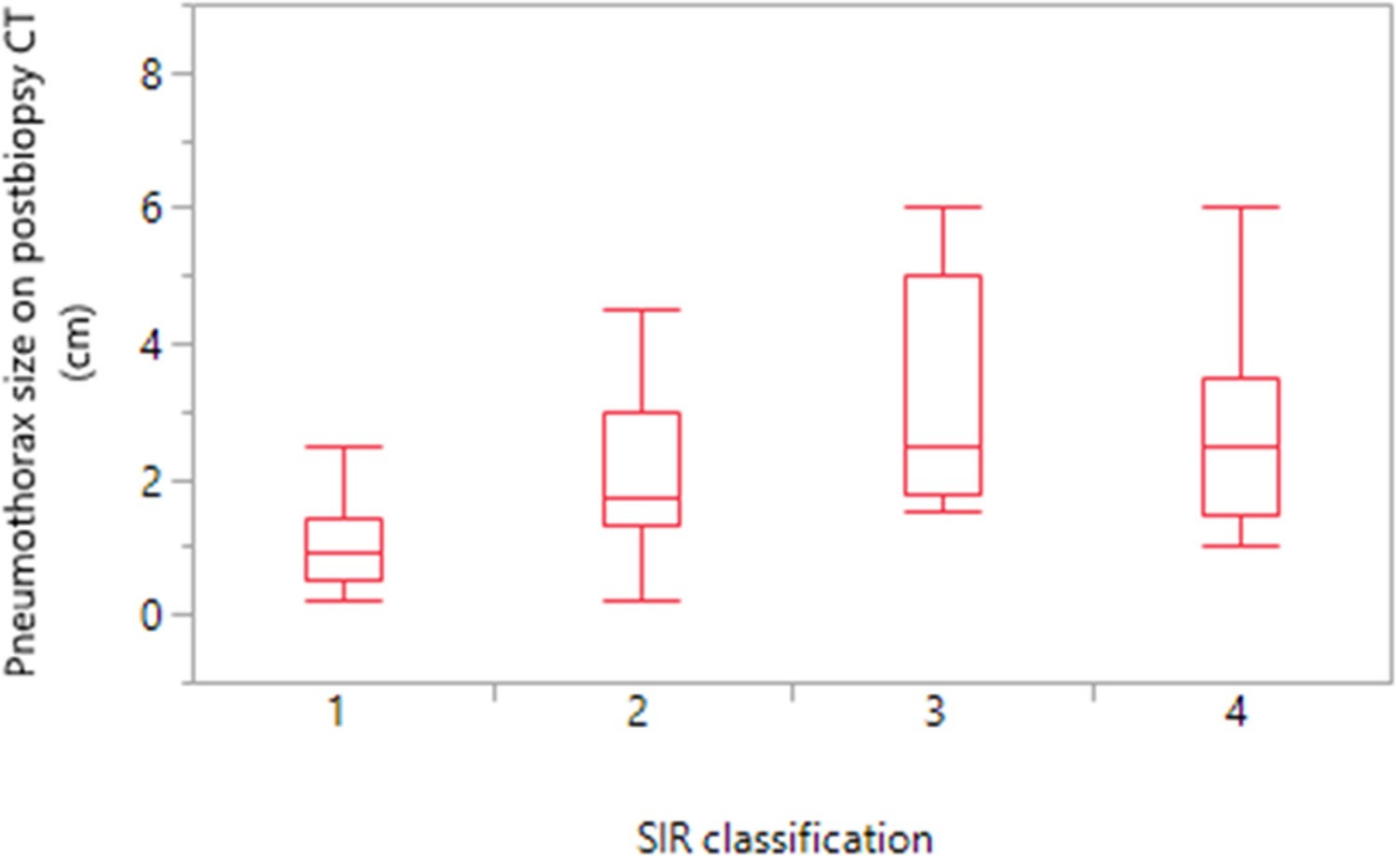
Pneumothorax size on post-biopsy CT images in relation to SIR classification. Box plots in the study group (n = 311).

There were 206 complications in 167 patients (53.6%). The complications were minor SIR1-2 in 142 patients (45.6%), and major SIR3-4 in 25 patients (8%). In our study, SIR5-6 complications were not present. The complications were pneumothorax and/or pulmonary hemorrhage (Figs 1 and 2). Pneumothorax was detected in 137 patients (44%). In 135/137 patients (98.5%), pneumothorax was detected on immediate post-biopsy CT images, and detected in two patients on follow-up chest radiographs >2h after biopsy. Pneumothorax size on post-biopsy CT increased with increasing the SIR complications (ANOVA p<0.001) (Fig 3). The mean size of pneumothorax on post-biopsy CT was 1.0cm (95%CI: 0.9–1.1 cm) in SIR1-2, and 3.3cm (95%CI: 1.9–4.6 cm) in SIR3-4. Grade 1 pulmonary hemorrhage was detected in 44 patients (14%) and grade 2 in 25 patients (8%); all were self-limited with no need of therapy. No documented post-biopsy hemoptysis in our data base was found. No other complications were detected.

Chest tube drainage was needed in 25 patients (18.2% of pneumothorax; 8% of all biopsies); in all of them, pneumothorax was detected on immediate post-biopsy CT images. Drainage catheters were placed for small pneumothorax (n = 6), and for large pneumothorax (n = 19). The mean duration of tube placement was 2.8 days (95%CI: 1.7–3.9 days). The time interval between the biopsy and drainage catheter placement was, either immediately after the biopsy (n = 5), <24h (n = 10), 24-48h (n = 9), or >48h (n = 1). Nineteen patients were admitted to the hospital for follow up of a large persistent or increased pneumothorax until the chest tube was inserted. One patient developed immediate post-biopsy small asymptomatic pneumothorax (1.5cm) which remained in size on follow-up radiographs up to 8 hours after biopsy, he was discharged and returned to the hospital >48h after biopsy with a large pneumothorax (5cm) causing symptoms of dyspnea, cough and chest pain requiring chest tube drainage. The mean total period of hospitalization for management of post-biopsy pneumothorax requiring chest tube drainage until chest tube removal was 3.2 days (95%CI: 2.2–4.3 days).

Increased overall SIR complications was observed with underlying emphysema (Chi-square p = 0.001), decreasing lesion size (ANOVA p<0.001), increasing lesion depth from the pleura (ANOVA p<0.001), prolonged procedure time (ANOVA p = 0.02), increasing number of procedural CT images (ANOVA p = 0.001), increased number of pleural passes (Chi-square p<0.001), fissure puncture (Chi-square p<0.001), and needle-to-blood vessel angle (Chi-square p<0.001), see Table 2 for all ANOVA results and Table 3 for all chi-square tests. Results of the sensitivity analysis with ordinal logistic regression were consistent with these results, except that necessary assumptions (of proportional odds) were not fulfilled for some predictors (See S1 Table).

**Table 2:** ANOVA results of continuous parameters in relation to SIR classification (n = 311).

Parameter	SIR0 (n = 144)	SIR1 (n = 134)	SIR2 (n = 8)	SIR3 (n = 7)	SIR4 (n = 18)	p value
**Patient age** (y)	60.3(57.7–62.8)	63.7(61.1–66.4)	66.3(55.5–77.1)	69.0(57.4–80.5)	62.3(55.1–69.5)	0.25
**Lesion size** (cm)	3.8(3.5–4.2)	2.3(2.0–2.7)	2.7(1.2–4.2)	2.2(0.6–3.8)	2.3(1.3–3.3)	**<0.001**
**Lesion-to-pleura distance** (cm)	0.8(0.5–1.0)	2.2(1.9–2.4)	2.8(1.7–3.9)	4.2(3.1–5.4)	4.2(3.4–4.9)	**<0.001**
**Thoracic wall thickness** (cm)	4.0(3.7–4.2)	3.9(3.7–4.2)	4.2(3.1–5.2)	3.7(2.6–4.8)	4.4(3.7–5.0)	0.79
**Number of procedural CT images**	28.4(25.5–31.3)	34.5(31.5–37.5)	38.2(25.9–50.5)	47.1(34.0–60.2)	39.1(30.9–47.3)	**0.001**
**Procedure time length** (min)	15.1(14.0–16.2)	16.5(15.3–17.6)	18.2(13.5–22.9)	23.2(18.2–28.3)	16.1(13.0–19.3)	**0.02**

Mean (95% confidence interval CI).

**Table 3:** Chi-square tests of ordinal and nominal parameters in relation to SIR classification (n = 311).

Parameter	SIR0 (n = 144)	SIR1 (n = 134)	SIR2 (n = 8)	SIR3 (n = 7)	SIR4 (n = 18)	p value
**Sex**						0.72
Male	85(46%)	76(41%)	5(3%)	5(3%)	13(7%)	
Female	59(47%)	58(46%)	3(2%)	2(1%)	5(4%)	
**Emphysema**						**0.001**
Yes	32(49%)	18(28%)	2(3%)	3(5%)	10(15%)	
No	112(46%)	116(47%)	6(2%)	4(2%)	8(3%)	
**Lesion lobar location**						0.21
Rt upper	25(42%)	24(40%)	1(2%)	1(2%)	8(14%)	
Rt lower	40(46%)	40(46%)	3(3%)	2(2%)	3(3%)	
Rt middle	8(33%)	15(63%)	1(4%)	0(0%)	0(0%)	
Lt upper	35(44%)	34(43%)	3(4%)	2(3%)	5(6%)	
Lt lower	36(59%)	21(35%)	0(0%)	2(3%)	2(3%)	
**Lesion morphologic characteristic**						0.05
Solid	106(43%)	110(45%)	5(2%)	6(3%)	17(7%)	
Subsolid	32(67%)	13(27%)	2(4%)	0(0%)	1(2%)	
Consolidative	4(40%)	6(60%)	0(0%)	0(0%)	0(0%)	
Cavitary	2(22%)	5(56%)	1(11%)	1(11%)	0(0%)	
**Lesion histopathology**						0.47
Malignant	93(45%)	93(45%)	7(3%)	4(2%)	10(5%)	
Benign	32(50%)	28(44%)	0(0%)	2(3%)	2(3%)	
Infection	10(50%)	7(35%)	0(0%)	0(0%)	3(15%)	
Non-diagnostic	5(33%)	6(40%)	1(7%)	1(7%)	2(13%)	
**Patient position**						0.90
Supine	58(45%)	59(46%)	4(3%)	2(2%)	6(5%)	
Prone	75(48%)	62(39%)	4(3%)	4(3%)	11(7%)	
Lateral	11(42%)	13(50%)	0(0%)	1(4%)	1(4%)	
**Number of pleural passes**						**<0.001**
Once	66(64%)	28(27%)	2(2%)	1(1%)	6(6%)	
Twice	61(48%)	58(46%)	2(2%)	0(0%)	5(4%)	
3 times	16(26%)	34(57%)	3(5%)	4(7%)	3(5%)	
4–5 times	1(5%)	14(63%)	1(5%)	2(9%)	4(18%)	
**Fissure puncture**						**<0.001**
Yes	1(7%)	7(43%)	1(7%)	3(21.5%)	2(21.5%)	
No	143(48%)	127(43%)	7(2%)	4(1%)	16(6%)	
**Needle-to-blood vessel angle**						**<0.001**
No angle	144(48%)	127(42%)	7(3%)	4(1%)	17(6%)	
Angle ≤90°	0(0%)	7(58.4%)	1(8.3%)	3(25%)	1(8.3%)	

Frequency (% of respective row).

A Univariate logistic regression model (no and minor complications SIR 0–2 vs. major complications SIR3-4) was done (Table 4) and results were compared to the previous analysis with ANOVA and chi-square tests, even though lesion size and procedure time were no longer significant: The risk for major complications increased with underlying emphysema (OR = 4.4, p<0.001), increasing lesion depth from the pleura (OR = 1.9 per cm, p<0.001), increasing number of procedural CT images (OR = 1.2 per 10 images, p = 0.01), increased number of pleural passes (OR up to 5 for worst category, p = 0.008), fissure puncture (OR = 10.3, p<0.001), and needle-to-blood vessel angle (OR = 6.2, p = 0.005).

**Table 4:** Univariate logistic regression analysis (no and minor complications SIR0-2 vs. major complications SIR3-4) of all variables in the study group (n = 311).

Variable	Comparator	OR	OR Upper CI	OR Lower CI	p value
**Age**	(per 10 years)	1.1	0.8	1.4	0.46
**Sex**	male	1.9	0.8	4.8	0.13
(ref = female)					
**Emphysema**	yes	4.4	1.9	10.2	**<0.001**
(ref = no)					
**Lesion size**	(per cm)	0.8	0.6	1.0	0.11
**Lesion lobar location**	rt upper	2.7	0.8	8.8	0.09
(ref = lt lower)	rt lower	0.8	0.2	3.0	0.79
	rt middle	0.2	0.01	5.3	0.38
	lt upper	1.3	0.3	4.5	0.65
**Lesion morphologic characteristic**	subsolid	0.2	0.05	1.5	0.14
(ref = solid)	consolidative	0.4	0.02	8.6	0.58
	cavitary	1.5	0.2	10.3	0.62
**Lesion-to-pleura distance**	(per cm)	1.9	1.5	2.4	**<0.001**
**Lesion histopathology**	malignant	0.9	0.3	2.6	0.90
(ref = benign)	infection	2.1	0.4	9.7	0.33
	non-diagnostic	3.0	0.6	14.2	0.16
**Patient position**	lateral	1.2	0.2	6.3	0.77
(ref = supine)	prone	1.7	0.7	4.1	0.22
**Thoracic wall thickness**	(per cm)	1.1	0.8	1.4	0.36
**Number of procedural CT images**	(per 10 images)	1.2	1.0	1.5	**0.01**
**Procedure time length**	(per 5 minutes)	1.2	0.9	1.6	0.06
**Number of pleural passes**	2x	0.5	0.1	1.8	0.34
(ref = 1x)	3x	2.1	0.7	6.1	0.17
	4-5x	5.1	1.5	17.2	**0.008**
**Fissure puncture**	yes	10.3	3.2	32.8	**<0.001**
(ref = no)					
**Needle-to-blood vessel angle**	angle≤90°	6.2	1.7	22.5	**0.005**
(ref = no angle)					

**Fig 4:**
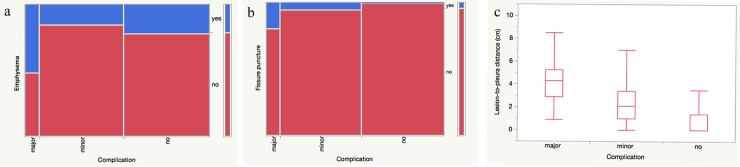
The independent risk factors for major complications in our study (n = 311). Mosaic plot of (a) emphysema and (b) fissure puncture in relation to SIR complications. (c) Box plot of lesion-to-pleura distance in relation to SIR complications.

A multiple regression model was made including emphysema, lesion-to-pleura distance, number of procedural CT images, number of pleural passes, fissure puncture and needle-to-blood vessel angle as highly significant univariate regression variables, as well as lesion size and procedure time length as clinically relevant variables (Table 5). In this model, we could demonstrate that emphysema (OR = 8.8, p<0.001), lesion depth from the pleura (OR = 1.9 per cm, p<0.001), and fissure puncture (OR = 9.4, p = 0.01) were the independent risk factors for major complications in our study (Fig 4).

**Table 5:** Multivariate logistic regression analysis (no and minor complications SIR0-2 vs. major complications SIR3-4) of selected variables in the study group (n = 311).

Variable	Comparator	OR	OR Upper CI	OR Lower CI	p value
**Emphysema** (ref = no)	yes	8.8	2.9	26.6	**<0.001**
**Lesion size**	(per cm)	0.8	0.6	1.1	0.31
**Lesion-to-pleura distance**	(per cm)	1.9	1.4	2.6	**<0.001**
**Number of procedural CT images**	(per 10 images)	1.0	0.7	1.5	0.76
**Procedure time length**	(per 5 minutes)	1.4	0.5	3.7	0.50
**Number of pleural passes** (ref = 1x)	2x	0.2	0.05	0.9	0.04
	3x	0.3	0.07	1.6	0.17
	4-5x	0.2	0.03	1.5	0.12
**Fissure puncture** (ref = no)	yes	9.4	1.5	57.0	**0.01**
**Needle-to-blood vessel angle** (ref = no angle)	angle≤90°	2.6	0.3	18.6	0.33

**Fig 5:**
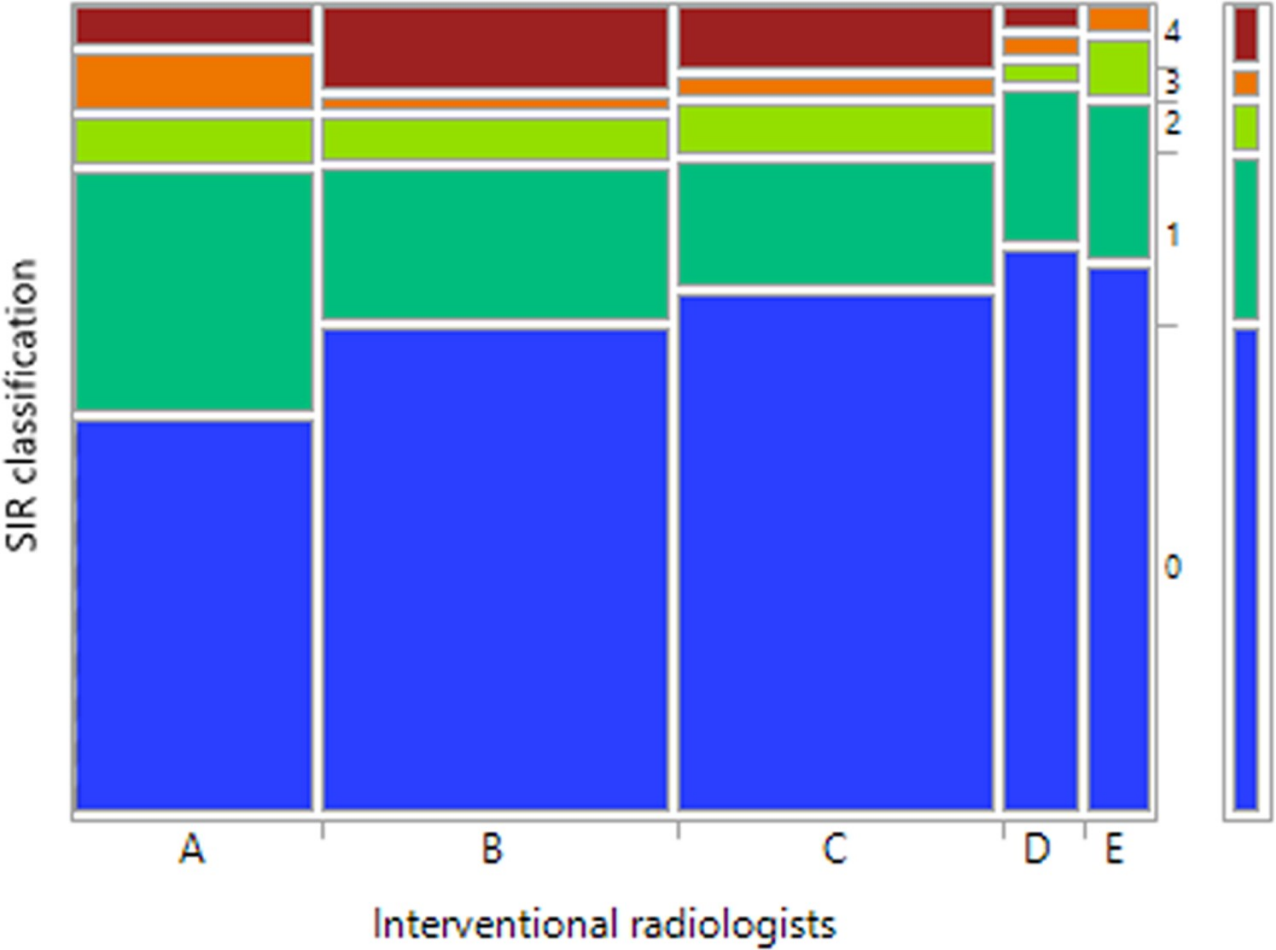
SIR classification in relation to interventional radiologists performing the biopsies. Mosaic plot in the study group (n = 311).

The biopsy procedures in the study population were performed by five interventional radiologists. We observed differences in the complication rates between the interventional radiologists performing biopsies (Fig 5), however they were not statistically significant (Chi-square p = 0.54).

## Discussion

In this study we could demonstrate that: a) CT-guided lung biopsy is a safe procedure. b) The overall SIR complication severity increased with underlying emphysema, smaller deeply located lesion, increased puncture time and number of procedural CT images, multiple pleural passes and fissure puncture. c) Emphysema, lesion depth from pleura, and fissure puncture are the independent risk factors for major complications in a multiple logistic regression model.

CT-guided lung biopsy is a safe technique for diagnosis of pulmonary lesions [2]. Due to low rate of complications, it may be performed on an outpatient basis with low rates of hospitalization [16], 25 patients (8%) in our study require therapy and variable periods of hospital stay. Few reports of life threatening complications such as tumor cell seeding along the needle tract, systemic air embolism and pericardial tamponade exist [17], however, none of these complications are present in our study.

In agreement with previously published studies, this study showed that pneumothorax and pulmonary hemorrhage were the most frequent reported complications of CT-guided lung biopsy [12, 18–20, 6]. The rates of pneumothorax in the literature vary widely. SIR and ACR published an estimated pneumothorax rate of 12–45% and a chest tube placement rate of 2–15% [3]. 44% of biopsies in our study are complicated by pneumothorax and 8% require chest tube drainage, which are acceptable comparable to previous studies. Pulmonary hemorrhage was less frequent with reported frequencies ranging from 4–27% [21, 22].This is also in a line with our findings, 22% of biopsies in our study are complicated by pulmonary hemorrhage (grade 1 pulmonary hemorrhage in 14% and grade 2 in 8%); none of them shows progression to a major complication. Hemoptysis was reported to occur in up to 20% of procedures [12], however, no documented cases of hemoptysis after biopsy in our data base is recorded. This can be explained as suggested by Tai et al. [13]: hemoptysis limited to hemorrhagic sputum may have been underreported. Furthermore, the concordance between the occurrence of significant pulmonary hemorrhage (grade ≥2) and hemoptysis is poor [12].

Many previous studies reported complications after CT-guided lung biopsy. Our study used the SIR classification to classify the severity of complications into minor complications SIR1-2, consisted in our study of pulmonary hemorrhage and/or pneumothorax without need of intervention, and major complications SIR3-4, consisted of pneumothorax requiring intervention. The overall SIR complication severity increased with underlying emphysema, smaller deeply located lesion, increased puncture time and procedural CT images, multiple pleural passes and fissure puncture.

In order to build a multiple regression model to assess the independent risk factors for major complications, a univariate logistic regression analysis is performed in relation to SIR score. Based on a multivariate logistic regression, this study clearly shows that emphysema, increased lesion depth from the pleura, and fissure puncture are the independent risk factors for major complications after CT-guided lung biopsy using a semi-automated 18 gauge biopsy system. Emphysema is a controversially discussed predictor for post-biopsy complications [4, 23]. Our study shows that emphysema increased the risk for major complications after biopsy. The association between trans-pulmonary needle path length and complication severity is in keeping with previous reports; the longer is the needle path from the pleural surface to the lesion, the more difficult accurate targeting of the needle with increased chances of pleural and lung parenchymal damage [4, 24, 9, 25, 26, 11, 7]. In comparison, biopsies of subpleural lesions in our study are not associated with occurrence of major complications, however, they may be associated with minor complications, such as small pneumothorax due to pleural injury, and/or pulmonary hemorrhage may be due to extension of the cutting needle into the normal surrounding lung parenchyma [12]. In our study, fissure puncture is also an independent risk factor for major complications. This could be explained by the transgression of many pleural surfaces by the biopsy needle [11]. Based on our study results, to minimize the risk of major complications after biopsy, the physicians should adopt the shortest possible trans-pulmonary needle path to the lesion and avoid fissure puncture.

We observe that no angle vs. angle ≤90° of the biopsy needle trajectory to a large blood vessel distal to the lesion, may be a significant risk factor (OR = 6.2, p = 0.005) for complication severity in univariate logistic regression analysis. However, p value of proportional-odds assumption in ordinal regression analysis is violated, which may be explained by the small number of biopsies with angle ≤90° (n = 12), in comparison to no angle (n = 299), making a valid and robust comparison difficult.

It is also important to point out that some variables are not related to the occurrence of overall or major complications in our study, e.g. age, sex, position of the patient during procedure, lesion lobar location, and thickness of thoracic wall at needle path. We observe a borderline p value of lesion morphologic characteristic in relation to SIR classification (Chi-square p = 0.05), but it shows insignificant p values in univariate logistic regression analysis and the test for assumption of its proportional odds ratios in univariate ordinal regression analysis is non-applicable, which may be explained by unfavorable distribution of the categories of a variable (large number of solid lesions (n = 244) in comparison to quite small groups of other morphological types).

Our study does not find a correlation between lesion histopathology with the risk of complications. However, a non-specific benign result does not always exclude malignancy, and further evaluation is required. Clinical and radiographic follow-up are essential, if further lesion growth occurs after a non-specific benign diagnosis is obtained with biopsy, repetition of biopsy or resection may be indicated [20, 27].

We believe that the rate of post-biopsy complications may be affected by experience and skills of the interventional radiologists performing the biopsies [19]. We observe differences in complication rates between interventional radiologists; however, they were not statistically significant. More studies are required to prove possible significant differences in rates of complications between different interventional radiologists.

There are some limitations to this study. First, as this study has a retrospective design, it is limited by patients who have already been selected to undergo biopsies, and by information already available in the database. Second, some patients who had developed post-biopsy complications were already hospitalized due to other causes rather than post-biopsy compilations, in such cases only management of post-biopsy complications are taken into consideration on the basis of retrospective review. Third, it is difficult to identify cases complicated with hemoptysis after biopsy in our study as post-biopsy hemoptysis is not documented systematically in our database.

## Conclusions

CT-guided lung biopsy is a safe procedure. Emphysema, lesion depth from the pleura, and fissure puncture are the independent risk factors for major complications after lung biopsy with a non-coaxial semi-automated 18 gauge biopsy system. In addition, smaller lesion, increased procedure time and number of procedural CT images, as well as multiple pleural passes are significant univariate risk factors for overall complication severity. Knowledge about risk factors influencing complication severity is important for planning and performing CT-guided lung biopsies.

## Supporting information

S1 Table Univariate ordinal regression analysis (SIR0-4) of all variables in the study group (n = 311). (DOCX)
