# Supplementary material for: Complications of CT-guided lung biopsy with a non-coaxial semi-automated 18 gauge biopsy system: Frequency, severity and risk factors
Source: PLoS One. 2019 Mar 18;14(3):e0213990. doi: 10.1371/journal.pone.0213990 (PMC6422294; doi:10.1371/journal.pone.0213990)
Supplement: S1 Table — (DOCX) [file pone.0213990.s001.docx]

**Supporting Information**

**S1 table: Univariate ordinal regression analysis (SIR0-4) of all variables in the study group (n=311).**

| **Variable** | **Comparator** | **OR** | **OR Upper CI** | **OR Lower CI** | **p value Regression** | **p value Prop Odds*** |
| --- | --- | --- | --- | --- | --- | --- |
|  |  |  |  |  |  |  |
| **Age** | (per 10 years) | 1.1 | 1.0 | 1.3 | 0.04 | <0.001 |
| **Sex** | male | 1.1 | 0.7 | 1.7 | 0.60 | 0.49 |
| (ref=female) |  |  |  |  |  |  |
| **Emphysema** | yes | 1.2 | 0.7 | 2.0 | 0.41 | 0.001 |
| (ref=no) |  |  |  |  |  |  |
| **Lesion size** | (per cm) | 0.7 | 0.6 | 0.8 | <0.001 | 0.07 |
| **Lesion lobar location** | rt upper | 2.3 | 1.1 | 4.6 | 0.01 | <0.001 |
| (ref=lt lower) | rt lower | 1.6 | 0.8 | 3.1 | 0.12 |  |
|  | rt middle | 2.1 | 0.8 | 5.3 | 0.10 |  |
|  | lt upper | 1.8 | 0.9 | 3.5 | 0.06 |  |
| **Lesion morphologic characteristic** | subsolid | 0.4 | 0.1 | 1.6 | 0.22 | NA |
| (ref=solid) | consolidative | 0.1 | 0.05 | 0.7 | 0.01 |  |
|  | cavitary | 0.4 | 0.07 | 2.2 | 0.30 |  |
| **Lesion-to-pleura distance** | (per cm) | 1.9 | 1.7 | 2.2 | <0.001 | 0.50 |
| **Lesion histopathology** | malignant | 1.2 | 0.7 | 2.0 | 0.48 | NA |
| (ref= benign) | infection | 1.1 | 0.4 | 3.0 | 0.77 |  |
|  | non-diagnostic | 2.5 | 0.8 | 7.3 | 0.08 |  |
| **Patient position** | lateral | 1.0 | 0.4 | 2.3 | 0.88 | 0.58 |
| (ref=supine) | prone | 0.9 | 0.6 | 1.5 | 0.90 |  |
| **Thoracic wall thickness** | (per cm) | 1.0 | 0.9 | 1.1 | 0.68 | <0.001 |
| **Number of procedural CT images** | (per 10 images) | 1.2 | 1.1 | 1.4 | <0.001 | 0.72 |
| **Procedure time length** | (per 5 minutes) | 1.2 | 1.0 | 1.4 | 0.01 | 0.01 |
| **Number of pleural passes** |  |  |  |  |  | <0.001 |
| (ref=1x) | 2x | 1.7 | 1.0 | 2.8 | <0.001 |  |
|  | 3x | 4.5 | 2.3 | 8.5 | <0.001 |  |
|  | 4-5x | 12.1 | 4.8 | 30.6 | <0.001 |  |
| **Fissure puncture** | yes | 8.8 | 3.1 | 24.6 | <0.001 | 0.32 |
| (ref=no) |  |  |  |  |  |  |
|  |  |  |  |  |  |  |
| **Needle-to-blood vessel angle** (ref=no angle) | angle≤90° | 7.0 | 2.3 | 20.9 | <0.001 | 0.02 |

* P value from test of proportional-odds assumption (if <0.05, assumption of proportional odds is violated).

N/A: non-applicable.
